# Evidence that Doublecortin Is Dispensable for the Development of Adult Born Neurons in Mice

**DOI:** 10.1371/journal.pone.0062693

**Published:** 2013-05-07

**Authors:** Katharina Merz, D. Chichung Lie

**Affiliations:** 1 Research Group Adult Neurogenesis and Neural Stem Cells, Institute of Developmental Genetics, Helmholtz Zentrum München, German Research Center for Environmental Health, Munich-Neuherberg, Germany; 2 Institute of Biochemistry, Emil Fischer Center, University of Erlangen-Nürnberg, Erlangen, Germany; Charité-Universitätsmedizin Berlin, Germany

## Abstract

In mammals, adult neural stem cells give rise to new hippocampal dentate granule neurons and interneurons of the olfactory bulb throughout life. The microtubule associated protein Doublecortin (DCX) is expressed by migrating neuroblasts and immature neurons, and is widely used as a stage-specific marker of adult neurogenesis and as a marker to identify neurogenic activity in the adult brain per se. Mutations in the *DCX* gene have been causally linked to human lissencephalic syndromes. Moreover, embryonic loss of DCX function interferes with neuronal migration and dendritic patterning in a species- and region-specific manner. A putative function of DCX in adult neurogenesis has not been directly explored. Here we show that overexpression of DCX in newly generated dentate granule neurons of the adult mouse brain has no effect on morphological maturation or migration. We also show that micro (mi) RNA-mediated retroviral knockdown of DCX does not alter morphological maturation of adult born dentate granule cells or migration of new neurons in either adult neurogenic niche. Thus, the present data indicate that DCX is dispensable for the development of new neurons in adult mice.

## Introduction

Neural stem cells give rise to new neurons in the subgranular zone of the hippocampal dentate gyrus (DG) and the subventricular zone (SVZ) of the lateral ventricle throughout life. The generation of a mature neuron involves a stereotypic sequence of developmental steps including proliferation, cell cycle exit, neuronal fate determination, maturation and functional integration into the pre-existing neural circuit. These developmental stages can be distinguished on the basis of the expression of stage-specific marker proteins [1].

Doublecortin (DCX) is a microtubule binding protein. The doublecortin (DCX) superfamily consists of 11 conserved members [2], all containing a DCX domain, which is necessary for microtubule binding [3]. DCX is highly expressed in migrating neurons of the developing central nervous system [4], [5], [6]. In the adult mouse brain, DCX is almost exclusively expressed by immature newborn neurons in the DG and the SVZ/OB-system and is commonly used to distinguish immature neurons from non-neuronally committed precursors and mature neurons, and to estimate neurogenic activity [7], [8], [9].

Mutations in the X-linked *Dcx* gene are associated with abnormal neuronal migration, and are causally linked to epilepsy, mental retardation, lissencephaly in male and subcortical laminar heterotopia in female human subjects [4], [10], [11]. Interestingly, there are species specific requirements for DCX function in the development of distinct forebrain regions. In humans, DCX is required for the lamination of the hippocampus and the neocortex [12]; in mice, only the lamination of the hippocampus is dependent on DCX function. RNAi-mediated knockdown of DCX causes heterotopia formation in the rat neocortex [13] but not in the murine neocortex [14]. Short-hairpin (sh) RNA-mediated DCX knockdown in the early postnatal SVZ/OB system of mice causes abnormal neuronal migration and changes the fate of developing neurons [15]. Despite the widespread use of DCX as a marker for immature neurons in the adult neurogenic lineage, little is known about the specific function of DCX in adult neurogenesis. Analysis of DCX null mutant mice suggested that DCX is required for the migration of adult-born neurons in the SVZ/OB-system [16]. DCX null mutant mice, however, lack DCX function already during embryonic development and thus do not allow to distinguish whether the observed migratory defects result from a direct function of DCX in adult-born neurons or result from defective CNS development. Here, we employ a MMLV-retrovirus based approach to overexpress or knockdown DCX specifically in the neurogenic lineage of the DG and the SVZ/OB-system during adulthood. Our results provide strong evidence that DCX is dispensable for the development of adult born neurons in wildtype mice.

## Materials and Methods

### Animals

All animal experiments were performed in accordance with the European Communities Council Directive (86/609/EEC). Stereotactic injections of retroviruses into the brain of adult mice were approved by the Government of Upper Bavaria. For all experiments, seven weeks old female C57BL/6-J mice were ordered from Charles River and retrovirally injected at an age of eight weeks. Mice were grouped housed in big rat cages under a 12 h light/dark cycle and had *ad libitum* access to food and water. Cages were containing a house and a running wheel.

### Vector Construction

For mouse moloney retrovirus (MMLV) -mediated expression of DCX, the cDNA of the murine DCX (oligos # 796, # 795; Table 1) was tagged with FLAG (3x) and cloned into CAG-IRES-GFP [17] or CAG-IRES-RFP to generate CAG-DCX-3xFLAG-IRES-GFP or CAG-DCX-3xFLAG-IRES-RFP. The CAG-RFP retrovirus has been described previously [17], [18]. pcDNA^TM^6.2-GW/EmGFP-miR from Invitrogen was filled with a linker (# 1015, # 1016) to generate two BsmBI restriction sites and restriction sites (BglII, AvrII, XhoI, MfeI, HindIII) unique in the CAG-vectors. The generated vector was named pcDNA6.2-GW-EmGFP-filled. EmGFP-miR-flanking cassette was PCR amplified (# 1025, # 1027) from pcDNA6.2-GW-EmGFP-filled and cloned into pKSSP (pBluescript KS modified with a SfiI site near the KpnI site and a PmeI site next to the SacII site; from Fred Gage, Salk Institute, La Jolla, USA) to generate pKSSP-EmGFP-miR. Three different miRNAs against DCX were constructed using BLOCK-iT™ RNAi Express from Invitrogen miDCX(1) (# 1072, # 1073), miDCX(2) (# 1074, # 1075) and miDCX(3) (# 1076, # 1077) and cloned into BsmBI sites of pKSSP-EmGFP-miR to generate pKSSP-EmGFP-miDCX. EmGFP-miDCX was subcloned into CAG-GFP by replacing GFP to generate CAG-EmGFP-miDCX. For construction of the internal control vector CAG-RFP-miCtr-IRES-RFP, EmGFP was replaced in pcDNA6.2-GW-EmGFP-filled by PCR amplified (# 1220, # 1221) RFP from CAG-RFP. RFP-miR-flanking cassette was PCR amplified (# 1222, # 1027) from pcDNA6.2-GW-RFP-filled and cloned into pKSSP to generate pKSSP-RFP-miR. As a control miRNA, the sequence (# 1170, # 1171) of pcDNA™6.2-GW/miR-neg control plasmid from Invitrogen was used and cloned into pKSSP-RFP-miR to generate pKSSP-RFP-miCtr. RFP-miCtr was subcloned into CAG-IRES-RFP to generate CAG-RFP-miCtr-IRES-RFP.

**Table 1:** Oligos used in this study.

oligo name	#	sequence 5′ –3′
DCX+1-for	796	GGGGCCGCCTGGGCCATGGAACTTGATTTTGGACA
DCX+1098-rev	795	GGGAATTCCATGGAATCGCCAAGTGA
miR_linker_for	1015	TGCTAGAGACGAGATCTCCTAGGCTCGAGCAATTGAAGCTTCGTCTCA
miR_linker_rev	1016	CCTGTGAGACGAAGCTTCAATTGCTCGAGCCTAGGAGATCTCGTCTCT
EmGFPmiRNA-for	1025	CAGGGCCGCCTCGGCCAATGGTGAGCAAGGGCGAGGAGCTG
miRNA-rev	1027	GTAGTTTAAACGGCCATTTGTTCCATGTGAGTGCT
miDCX(1)-for	1072	TGCTGATTACTTAATGCCTGCAAGGTGTTTTGGCCACTGACTGACACCTTGCACATTAAGTAAT
miDCX(1)-rev	1073	CCTGATTACTTAATGTGCAAGGTGTCAGTCAGTGGCCAAAACACCTTGCAGGCATTAAGTAATC
miDCX(2)-for	1074	TGCTGTGGAGTAGCACACTTTGAAGTGTTTTGGCCACTGACTGACACTTCAAAGTGCTACTCCA
miDCX(2)-rev	1075	CCTGTGGAGTAGCACTTTGAAGTGTCAGTCAGTGGCCAAAACACTTCAAAGTGTGCTACTCCAC
miDCX(3)-for	1076	TGCTGATTTCTAGATGCTTTGTCTCGGTTTTGGCCACTGACTGACCGAGACAACATCTAGAAAT
miDCX(3)-rev	1077	CCTGATTTCTAGATGTTGTCTCGGTCAGTCAGTGGCCAAAACCGAGACAAAGCATCTAGAAATC
miR-neg-for	1170	TGCTGAAATGTACTGCGCGTGGAGACGTTTTGGCCACTGACTGACGTCTCCACGCAGTACATTT
miR-neg-rev	1171	CCTGAAATGTACTGCGCGTGGAGACGTCAGTCAGTGGCCAAAACGTCTCCACGCAGTACATTTC
RFP+1-DraI-for	1220	GGCTTTAAAATGGCCTCCTCCGAGGACGTCATC
RFP+678-Dral-rev	1221	CCCTTTAAACGATCGACGGCCACGAAGTGCTTAGCTTAGGCGCCGGTGGAGTGGC
RFPmiRNA-for	1222	CAGGGCCGCCTCGGCCAATGGCCTCCTCCGAGGACGTCATC

Note: as the GFP signal in CAG-EmGFP-miDCX retrovirally transduced cells was not sufficient to study the dendrite morphology of transduced cells, CAG-EmGFP-miDCX-IRES-GFP was generated. Fluorescence signal was higher compared to IRES-less vector, but IRES-GFP containing vector was not resulting in an *in vivo* knockdown of DCX!

### Retrovirus Preparation

Retroviruses were produced from the plasmids above as described previously [19]. Viruses were harvested four times; first harvest was three days after transfection and the following ones every second day. The obtained viral titers ranged between 10^5^–10^8^ colony forming units (cfu)/ml, dependent on the harvest number and the construct. For CAG-EmGFP-miDCX viruses only low titers (<10^6^ cfu/ml) were obtained.

### Stereotactic Injections

All experiments were conducted under the condition that animals have ad libitum access to a running wheel during the whole duration of the experiment. This measure was necessary to enhance proliferation and thus the number of transduced cells in the context of the loss-of-function experiments, because of the low titer of the CAG-EmGFP-miDCX vectors. In order to be able to compare gain- and loss-of-function results, animals in the gain-of-function experiments also had free access to a running wheel.

Mice were deeply anesthetized with a mixture of fentanyl (0.05 mg/kg bodyweight), midazolam (5 mg/kg bodyweight) and medetomidine (0.5 mg/kg bodyweight). Anaesthesia was reverted with a mixture of buprenorphine (0.1 mg/kg bodyweight), atipamezole (2.5 mg/kg bodyweight) and flumazenil (0.5 mg/kg bodyweight). Mice were stereotactically injected with 0.9 µl of CAG-DCX-3xFLAG-IRES-GFP and CAG-RFP (titer 1.11×10^7^ cfu/ml each) or CAG-EmGFP-miDCX (estimated titer <10^6^ cfu/ml; precise titer could not been determined due to low GFP signal; undiluted usage) and CAG-RFP-miCtr-IRES-RFP (titer 1.11×10^7^ cfu/ml) retroviruses, into the left and right dentate gyrus (coordinates from bregma were −1.9 mm anterior/posterior, ±1.6 mm medial/lateral, −1.9 mm dorsal/ventral from dura) or into the left and right RMS (coordinates from bregma were +2.3 mm anterior/posterior, ±0.8 mm medial/lateral, −2.9 mm dorsal/ventral from dura). Group size was *n* = 4–5 mice per time point for dentate gyrus neurogenesis analysis, and *n* = 3 per time point for SVZ/OB neurogenesis.

### Tissue Processing

Mice were sacrificed with CO_2_ at the time points indicated. They were perfused for 5 min with PBS (pH 7.4), followed by 4% formaldehyde (FA) for 5 min. Brains were removed and postfixed in 4% FA over night at 4°C and were subsequently transferred to 30% sucrose in 0.1 M phosphate buffer. Forty, 80 or 120 µm-thick coronal brain sections were cut using a sliding microtome (Leica Microsystems). Brain sections were stored at −20°C in cryoprotect solution (0.05 M phosphate butter, 25% ethylenglycol v/v, 25% glycerol v/v).

### Immunhistochemistry and Phenotyping of Transduced Cells

Brain sections were washed three times in tris buffered saline (TBS) and blocked for 60 min in TBS supplemented with 3% normal donkey serum and 0.25% Triton X-100. Sections were incubated with primary antibodies diluted in blocking solution over one or two nights at 4°C. Primary antibodies against the following antigens were used: doublecortin (DCX; goat, 1∶250; Santa Cruz Biotechnology sc-8066), GFP (chicken, 1∶1.000; Aves Labs GFP-1020), RFP (rat, 1∶50; [20]), calretinin (rabbit, 1∶1.000; Swant 7699/4) and NeuN (mouse, 1∶50; hybridoma supernatant, Richard Mullen, University of Utah, Salt Lake City, UT). Sections were washed three times with TBS, blocked for 30 min and incubated with secondary antibodies, diluted 1∶250 in blocking solution for 2 h at room temperature. Secondary antibodies coupled to FITC, Cy3, Cy5 or CF633 (Biotium, 1∶500) from The Jackson Laboratory (Bar Harbor, USA) were previously resuspended in 400 µl water. After 10 min incubation with DAPI (10 mg/ml 4′,6-diamidino-2-phenylindole; 1∶10.000; Sigma-Aldrich) diluted in TBS, sections were washed twice in TBS and mounted in Aqua PolyMount (Polysciences). For phenotypical characterization of the retrovirally transduced cells, 40 µm sections containing the dentate gyrus or RMS were selected and immunstained. Selected sections from different animals were similar with regard to their position along the dorsoventral axis (for dentate gyrus analysis) and along the mediolateral axis (for RMS/OB analysis). Transduced cells were identified based on the expression of GFP and/or RFP. All transduced cells were analyzed by confocal microscopy for immunoreactivity for the respective marker (*n = *20–220 cells per animal and marker). Confocal single-plane images and *Z*-stacks were obtained on a Leica SP5 confocal microscope. Z-stacks were used for analysis of spines, dendritic morphology, phenotyping and migration analysis. Distance between single planes were 0.21 µm for spine analysis, 0.3 µm for dendritic morphology, and 1.5 µm for all other analyses.

### Morphology

For analysis of dendritic morphology, 120 µm thick sections were used to ensure that the section comprised the entire dendritic tree. For analysis of spines, 120 and 80 µm thick sections were used. RFP_only_-transduced control cells and yellow double transduced cells were morphologically analysed for the following parameters: complexity (Scholl analysis), number of branches, total dendritic length and number of spines per 10 µm. Only the RFP channel of confocal images of 2–6 RFP_only_ and yellow cells respectively per animal (*n* = 3) was analysed with Imaris7.2 (Bitplane).

### Migration in RMS/OB System

Images were taken from three different positions along the RMS/OB system: rostral part of the RMS (RMS1), middle part of the RMS (RMS2), and core region of the OB where new neurons switch their migratory mode from tangential to radial migration (see Figure 8E and Figure 4 in [15]). All virus-transduced cells in these positions were counted. Comparison of the ratio of yellow cells to all red cells (yellow+RFP_only_ cells) in these regions was performed to determine whether DCX manipulation altered positioning/migration along the RMS/OB system.

### Statistical Analysis

Unpaired Student’s t-test was used for analysis. Differences were considered statistically significant at p<0.05. All data are presented as mean ± s.d.

### Transfection and Western Blot

HEK 293T cells were co-transfected with the CAG-EmGFP-miDCX and the CAG-DCX-3xFLAG-IRES-RFP plasmids or alone with the CAG-DCX-3xFLAG-IRES-RFP plasmid as a negative control using the calcium phosphate method. 2.5 µg of DNA were transfected per plasmid. Proteins were extracted as described previously [21]. Nuclear and cytosolic extracts were pooled and 10 µg of whole cell extracts were analyzed by western blot.

Proteins were blotted on a PVDF membrane and blocked with 5% slim milk powder in PBS for 1h at room temperature (RT). Primary antibodies were diluted in a solution of 2% slim milk powder in PBST (0,05% Tween20 in PBS) and incubation was performed for 1 h at RT. Antibodies against the following antigens were used: FLAG (mouse, 1∶2.000; Sigma F1804) and β-actin (mouse, 1∶10.000; Abcam ab6276). Primary antibody incubation was performed under constant shaking for 1 h at RT. The membrane was washed three times with PBST and incubated with HRP-conjugated secondary antibodies diluted in 2% slim milk powder in PBST for 45 min at RT (mouse, 1∶10.000; The Jackson Laboratory 115-035-003). After three washes in PBST, blots were incubated with ECL solution (GE Healthcare). Chemiluminescence signals were detected with a CCD-camera (Fusion-SL 3500.WL; Peqlab) and proteins were quantified with the appropriate software (FusionCapt). FLAG signals were related to β-actin signals whereby DCX-3xFLAG single transfection signal was set to 1.

Both antibodies used in this study were raised in mice and the expected sizes were almost the same (∼50 kDa). As stripping of the blots for either FLAG or β-actin antibody was inefficient, it was impossible to hybridize one membrane with both antibodies. Therefore for each construct two blots were performed with the same amount of proteins and the same protein extracts.

## Results

**Figure 1:**
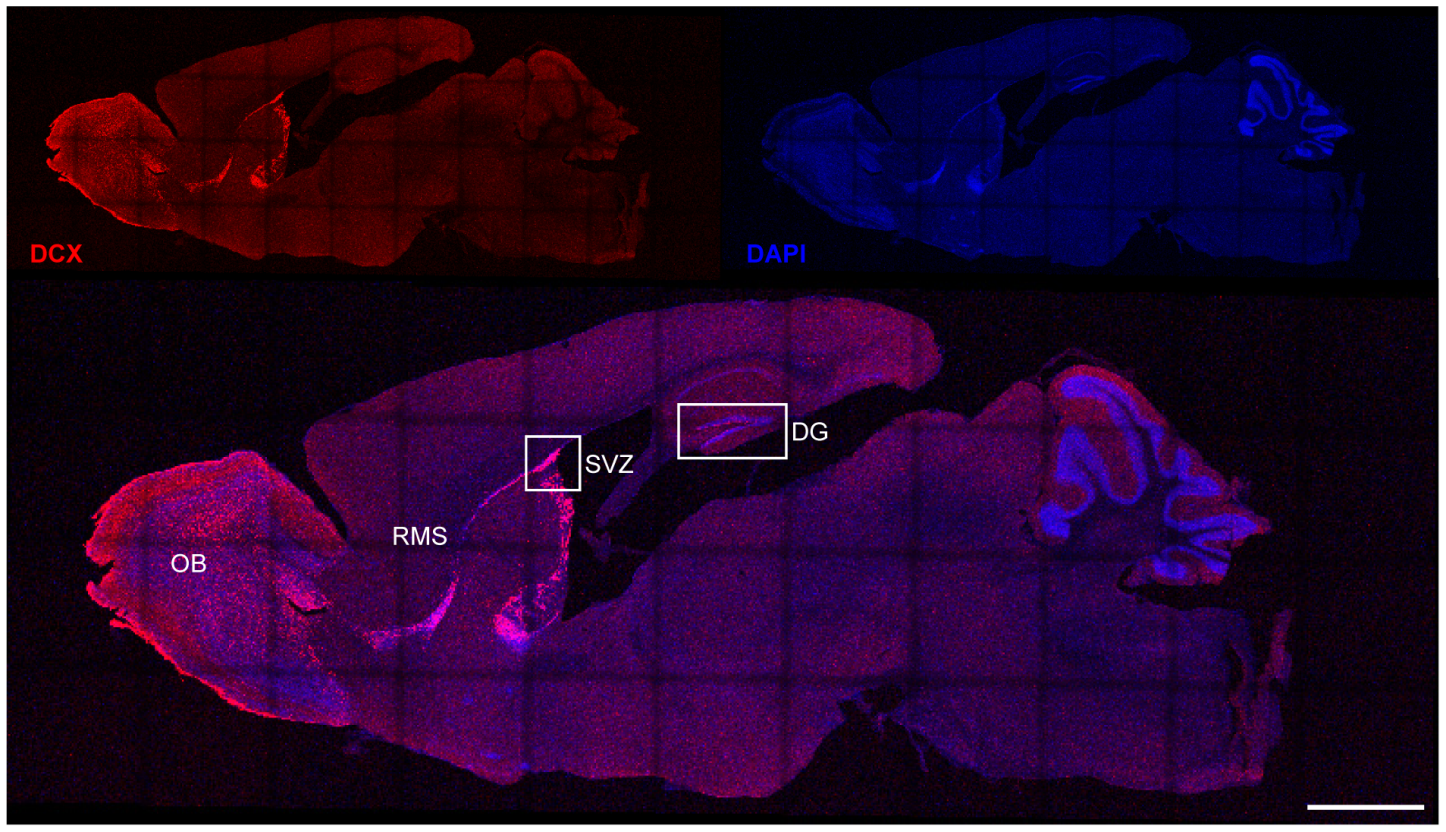
DCX expression in the adult mouse brain. DCX is almost exclusively expressed in immature neurons of the two neurogenic niches, the hippocampal dentate gyrus (DG) and the subventricular zone (SVZ). Neurons, born in the SVZ, are migrating along the rostral migratory stream (RMS) towards the olfactory bulb (OB). Scale bar 1250 µm.

DCX is almost exclusively expressed in immature neurons of the two neurogenic niches, the hippocampal dentate gyrus (DG) and the subventricular zone (SVZ; Figure 1). Some postmitotic cells of the piriform cortex express DCX. A subpopulation of these cells retains immature characteristics and is associated with synaptic plasticity [22]. The main goal of this study was to investigate the role of DCX in adult neurogenesis, where it is commonly used as a marker for the immature state of adult born neurons. To address this question we performed gain- and loss-of-function studies.

### Gain of Function

Previous work demonstrated that voluntary wheel running accelerates maturation of newborn dentate granule neurons as evidenced by morphological and electrophysiological criteria and an earlier transition of newborn neurons from a DCX-positive to a DCX-negative stage [23]. Consistent with this observation, we found that approximately 42% of newborn neurons in mice housed under standard conditions expressed DCX [17], whereas DCX expression was absent from newborn neurons at 28 dpi (days post injection), if animals have ad libitum access to a running wheel during the whole duration of the experiment.

**Figure 2:**
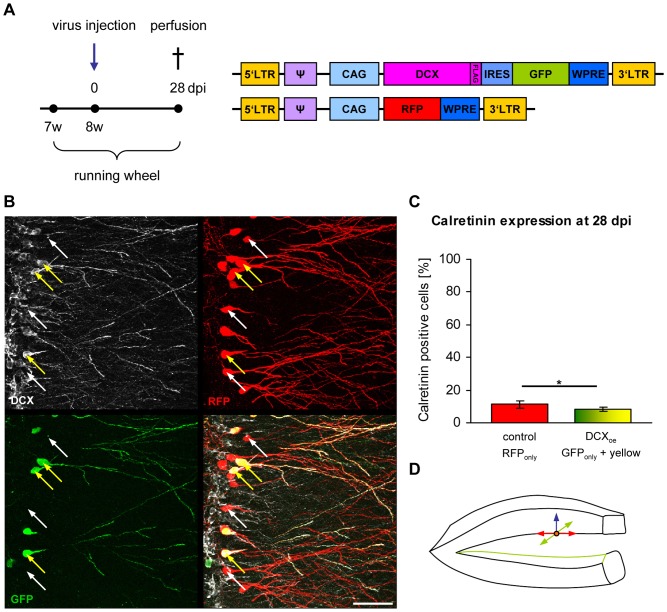
DCX overexpression has no effect on maturation and migration of newborn dentate granule cells at 28 dpi. (A) Experimental paradigm and retroviral constructs. (B) DCX overexpressing green or yellow cells are DCX positive (yellow arrows) whereas RFP_only_ control cells do not express DCX (white arrows). Scale bar 50 µm. (C) Quantification of calretinin expression in RFP_only_ control cells and in green and yellow DCX overexpressing cells (*p<0.05). (D) Newborn dentate granule cells can potentially migrate into three directions related to their place of birth in the subgranular layer: medial/lateral (red arrows), septal/temporal (green arrows) and perpendicular to these two directions through the granular layer (blue arrow). DCX expression does not alter perpendicular migration of the newborn neurons through the granular layer.

To investigate if cells are kept in an immature state by overexpression/prolonged expression of DCX, eight weeks old female C57/BL6 mice were injected with CAG-RFP and CAG-DCX-3xFLAG-IRES-GFP retroviruses. Animals were sacrificed at 28 dpi (Figure 2A). Three populations of retrovirally transduced cells were expected: single transduced cells with either CAG-RFP (red RFP_only_ cells) or CAG-DCX-3xFLAG-IRES-GFP (green GFP_only_ cells) or double transduced cells (red and green = yellow cells). RFP_only_ cells were used as an internal control and compared with GFP_only_ and/or yellow cells.

Immunstainings for DCX showed that, at 28 dpi, none of the RFP_only_ cells (white arrows) were positive for DCX, whereas all green cells (yellow arrows) were expressing DCX (Figure 2B). Brain sections were stained for calretinin, a calcium binding protein, which is expressed in immature dentate granule neurons [24]. 11.29% ±2.13% of the RFP_only_ control cells were positive for calretinin whereas 8.05% ±1.32% of DCX overexpressing green cells were calretinin positive (p = 0.04888412; Figure 2C), indicating that DCX overexpression does not maintain newly generated dentate granule neurons in a calretinin-positive immature state.

**Figure 3:**
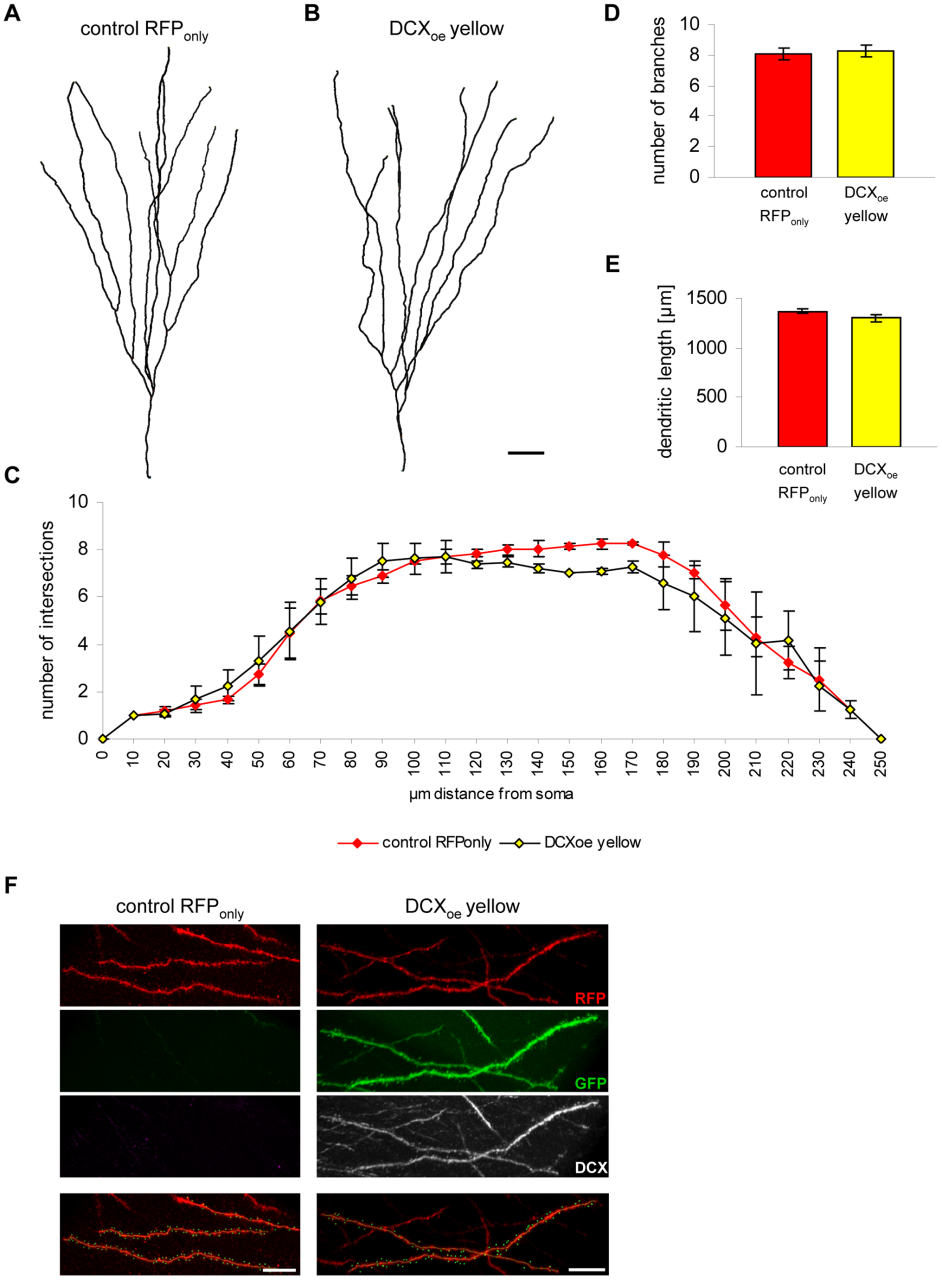
Prolonged DCX expression has no influence on morphological maturation of 28 days old dentate granule cells. (A–B) 3D reconstruction of an RFP_only_ (A) and a DCX-overexpressing yellow (B) granule neuron. Scale bar 20 µm. (C) Scholl analysis reveals no major changes in complexity in DCX overexpressing yellow cells compared to RFP_only_ control cells (*n* = 3 animals). (D–E) Quantification of the number of branch points and the total dendritic length in RFP_only_- and DCX-transduced yellow cells (*n* = 3 animals). (F) Spine analysis of an RFP_only_ and a DCX-overexpressing yellow cell. Green dots represent one spine. Scale bars 10 µm.

DCX knockout mice show branching defects in migrating interneurons [25]. To investigate if morphological maturation is influenced by DCX, dendrite complexity (via Scholl analysis; Figure 3A–C), number of branches (Figure 3D), total dendritic length (Figure 3E) and the number of spines per 10 µm (8.40±1.87 spines/10 µm in RFP_only_ cells and 8.14±0.13 spines/10 µm in yellow cells) were analysed (Figure 3F). None of these parameters were changed in DCX overexpressing cells compared to RFP expressing control cells.

Next, we investigated, whether migration of newborn cells in the DG is impaired by DCX overexpression. Under physiological circumstances, new dentate granule neurons migrate only 1–2 cell diameters from the SGZ towards the molecular cell layer. Around 80% of mature neurons are located in the inner part of the granular layer, 20% in the middle layer and only a small proportion of cells are located in the outer layer [26]. This short distance migration can be largely extended under pathological conditions such as knockdown of the schizophrenia susceptibility gene *Disc1* [27] or seizures [28], [29], [30], where newborn neurons can migrate even beyond the boundaries of the granule cell layer. DCX overexpressing green cells, however, were equally distributed like the RFP_only_ control cells (Figure 2B), strongly suggesting that DCX expression did not alter the migration of newborn neurons.

Taken together, our results indicate that prolonged expression/overexpression of the immature marker protein DCX does not influence maturation or migration of newly generated dentate granule neurons at 28 days post injection.

### Loss-of-function

DCX is required for the lamination of the hippocampus during development [12] and was suggested to be required for the migration of adult-born neurons in the SVZ/OB-system [16]. To address the question whether acute loss-of DCX function affects the maturation and/or migration of adult born cells in the DG or SVZ/OB-system of wildtype mice, a MMLV-retroviral expression vector was generated to knockdown DCX in neural stem cell progeny *in vivo*. Three different miRNAs against DCX were constructed using BLOCK-iT™ RNAi Express (Invitrogen).

**Figure 4:**
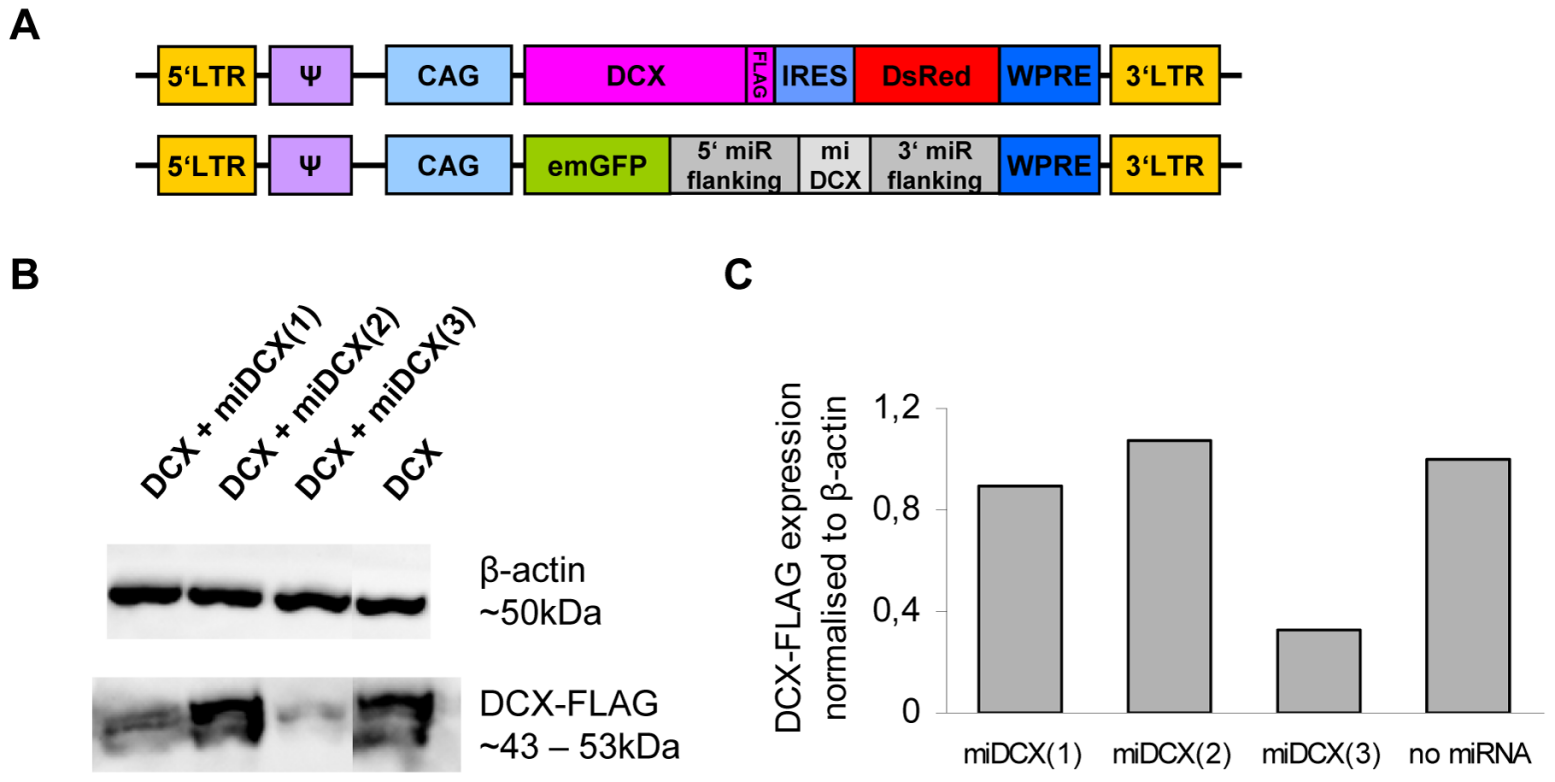
miDCX construct #3 knocks DCX efficiently down *in vitro*. (A) HEK 293T cells were co-transfected with the three different CAG-EmGFP-miDCX-constructs and the CAG-DCX-3xFLAG-IRES-RFP plasmid (B, lanes 1–3) or alone with the CAG-DCX-3xFLAG-IRES-RFP plasmid as a negative control (B, lane 4). (B) Western blots of whole cell extracts, hybridized with antibodies for FLAG-tag to visualize DCX-3xFLAG (expected size ∼43–53 kDa) and β-actin (∼50 kDa). (C) Quantification of the western blots. FLAG signals were normalised to β-actin signals whereby DCX-3xFLAG single transfection signal was set to 1.

**Figure 5:**
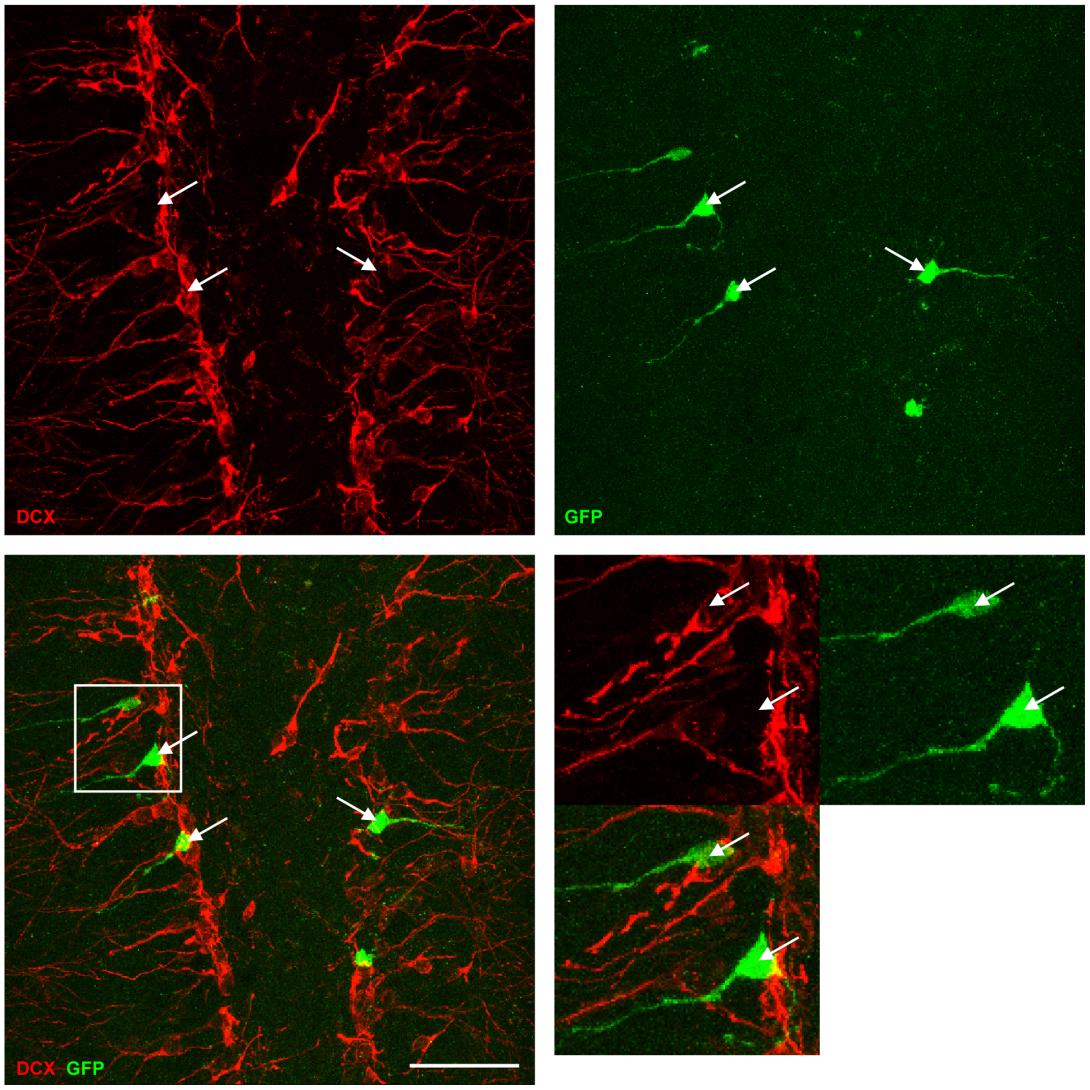
Efficient *in vivo* knockdown of DCX at 11 dpi. DCX is efficiently knocked down in GFP-miDCX(3)-transduced (green) neurons. Scale bar 50 µm.

Prior to virus production, knockdown efficiency was tested *in vitro* by co-transfection of HEK 293T cells with CAG-EmGFP-miDCX and CAG-DCX-3xFLAG-IRES-RFP and western blot against DCX (Figure 4). Constructs #1 and #3 were directed against a sequence located in the ORF while construct #2 was directed against a sequence in the 3′UTR of DCX. Consequently for construct #2, an *in vitro* knockdown of DCX was not expected as CAG-DCX-3xFLAG-IRES-RFP only contained the cDNA of DCX (Figure 4B–C, lane 2). Compared to CAG-DCX-3xFLAG-IRES-RFP only transfection, knockdown efficiency of construct #1 was 10.5%. Construct #3 showed much higher efficiency with a reduction of 66.9% in DCX expression (Figure 4C). To test if the miDCX vector was able to knockdown DCX *in vivo*, three female mice were injected with CAG-EmGFP-miDCX retrovirus for either construct #2 or #3 and analysed at 11 dpi (Figure 5). Both constructs were able to knock DCX down *in vivo*, whereby the efficiency of construct #3 was higher. Hence, construct #3 was used for subsequent *in vivo* loss-of-function studies.

**Figure 6:**
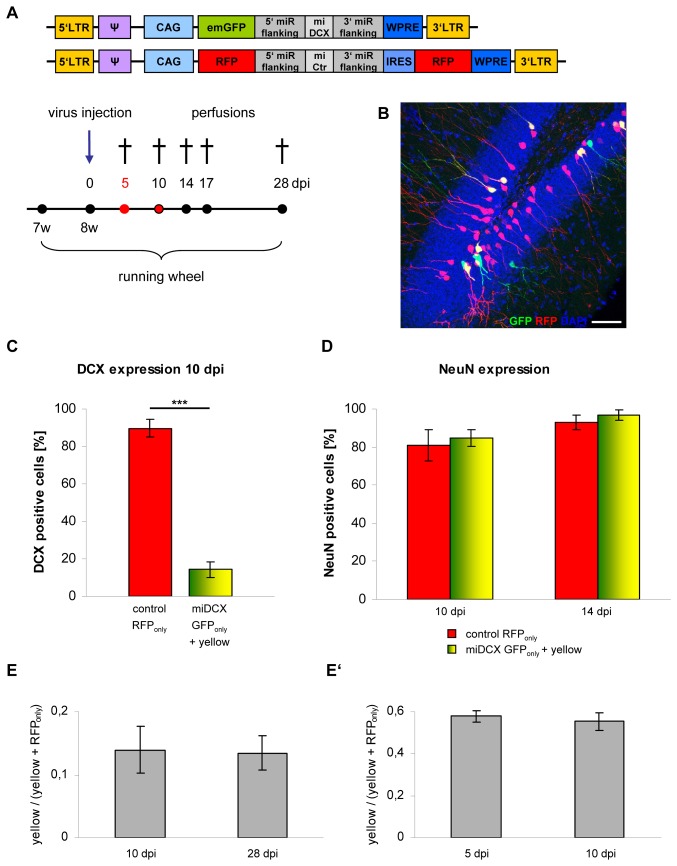
DCX knockdown does not affect maturation and survival rate of newborn neurons in the DG. (A) Experimental paradigm and retroviral constructs. (B) Distribution of green and yellow cells in the DG is comparable to RFP_only_ control cells. Scale bar 50 µm. (C) Quantification of DCX expression in RFP_only_ control cells compared to green and yellow miDCX-expressing cells. DCX is efficiently reduced in miDCX-expressing dentate granule cells at 10 dpi (***p<0.0005). (D) Quantification of NeuN expression in RFP_only_ control cells compared to green and yellow miDCX-expressing cells. NeuN, a marker for mature neurons is already at 10 dpi highly expressed in all transduced neurons (n.s. p>0.05). (E, E’) Survival rates of newborn cells, are not influenced by knockdown of DCX (n.s. p>0.05).

For maturation and survival analyses, five animals were injected with CAG-GFP-miDCX(3) and CAG-RFP-miCtr-IRES-RFP (internal control) retroviruses and sacrificed for analysis at the following time points: 10, 14, 17 and 28 dpi (Figure 6A). Knockdown efficiency was determined by DCX immunostainings (Figure 6C). While 89.6±4.87% of the RFP_only_ control cells were positive for DCX, only 14.32±4.06% of the GFP cells were positive for DCX with comparable signal intensity as the red control cells. The remaining GFP-labelled cells were either DCX negative or had a barely detectable DCX signal.

To test if maturation of the newborn cells is accelerated following DCX knockdown, immunostainings for the splicing factor NeuN, which is commonly used as a marker for mature neurons, were performed. At 10 dpi, 80% of the control cells were already NeuN positive (Figure 6D; in different experiments we detected in the meantime that roughly the same percentage of 5 dpi old cells were already NeuN positive; unpublished data). DCX knockdown did not significantly enhance the fraction of NeuN-expressing cells, demonstrating that loss-of-DCX does not promote the transition to a NeuN positive developmental stage.

Comparison of the position of DCX-knockdown cells and control cells, indicated that lack of DCX expression does not alter migration along the SGZ/molecular cell layer axis (Figures 6B, 2D).

**Figure 7:**
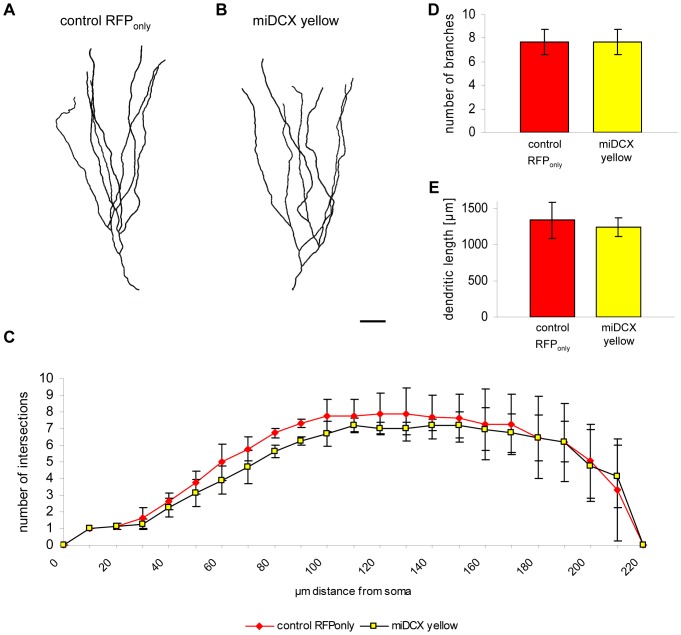
DCX knockdown has no effect on morphological maturation of newborn dentate granule neurons. (A–B) Imaris 3D reconstruction of an RFP_only_ (A) and a DCX-knockdown yellow (B) granule neuron. Scale bar 20 µm. (C) Scholl analysis reveals no changes in dendritic branching in DCX ablated yellow cells compared to RFP_only_ control cells (*n* = 3 animals). (D–E) Quantification of the number of branch points and the total dendritic length in RFP_only_- and miDCX-transduced yellow granule neurons (n.s. p>0.05; *n* = 3 animals).

DCX knockout mice display branching defects in migrating interneurons [25]. Analysis of 28 dpi cells, however, revealed no changes in complexity, the number of branches and the total dendritic length (Figure 7), indicating that acute loss-of-DCX does not alter the overall morphological development of new DG neurons.

Finally, we determined whether DCX is required for the survival of new DG neurons. Coinjection of the CAG-GFP-miDCX(3) virus together with the internal control virus CAG-RFP-miCtr-IRES-RFP for at least two different time points, enables the investigation of the influence of DCX knockdown on the number of newborn cells by comparison of the ratios of yellow cells to all red cells (yellow+RFP_only_ cells) at the different time points, independently of variations in the viral titers and injection sites [17], [31]. If survival of newborn cells would be affected, the ratio would decline. Survival rate of the DCX lacking cells was determined by comparison of the ratios at 10 dpi and 28 dpi (Figure 6E). Ratios did not significantly change suggesting that the survival rate of new neurons was not affected by DCX ablation. To exclude the possibility that DCX affected survival prior to 10 dpi, the experiment was preformed for the 5 dpi and 10 dpi (Figure 6E’; ratios between experiments can strongly vary due to different amounts of virus particles in the different injection mixes, therefore always two time points per survival assay are needed at least). Again, ratios between these time points were comparable, indicating that DCX knockdown did not affect survival of newly generated neurons.

**Figure 8:**
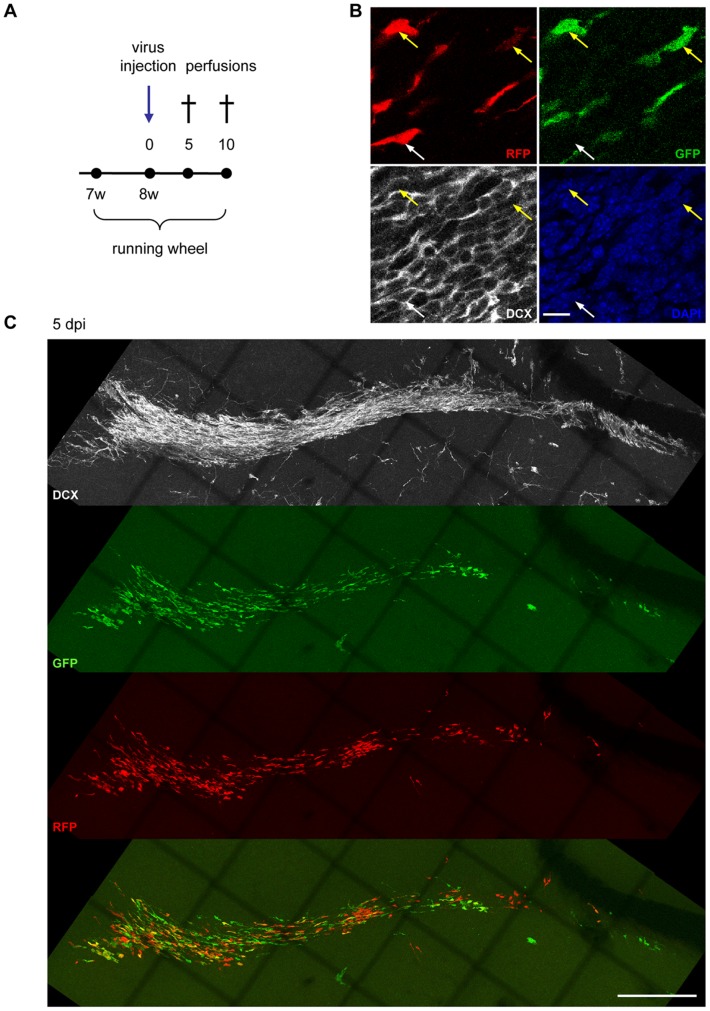
Migration of adult born neurons of the SVZ/OB-system is not altered 5 days after DCX knockdown. (A) Experimental paradigm. (B) Efficient *in vivo* knockdown of DCX in the SVZ of miDCX-transduced neurons (yellow arrows) at 10 dpi while RFP_only_ control cells (white arrow) express DCX. Scale bar 10 µm. (C) DCX ablated neurons (green and yellow) are migrating similar to RFP_only_ control cells at 5 dpi within the RMS.

**Figure 9:**
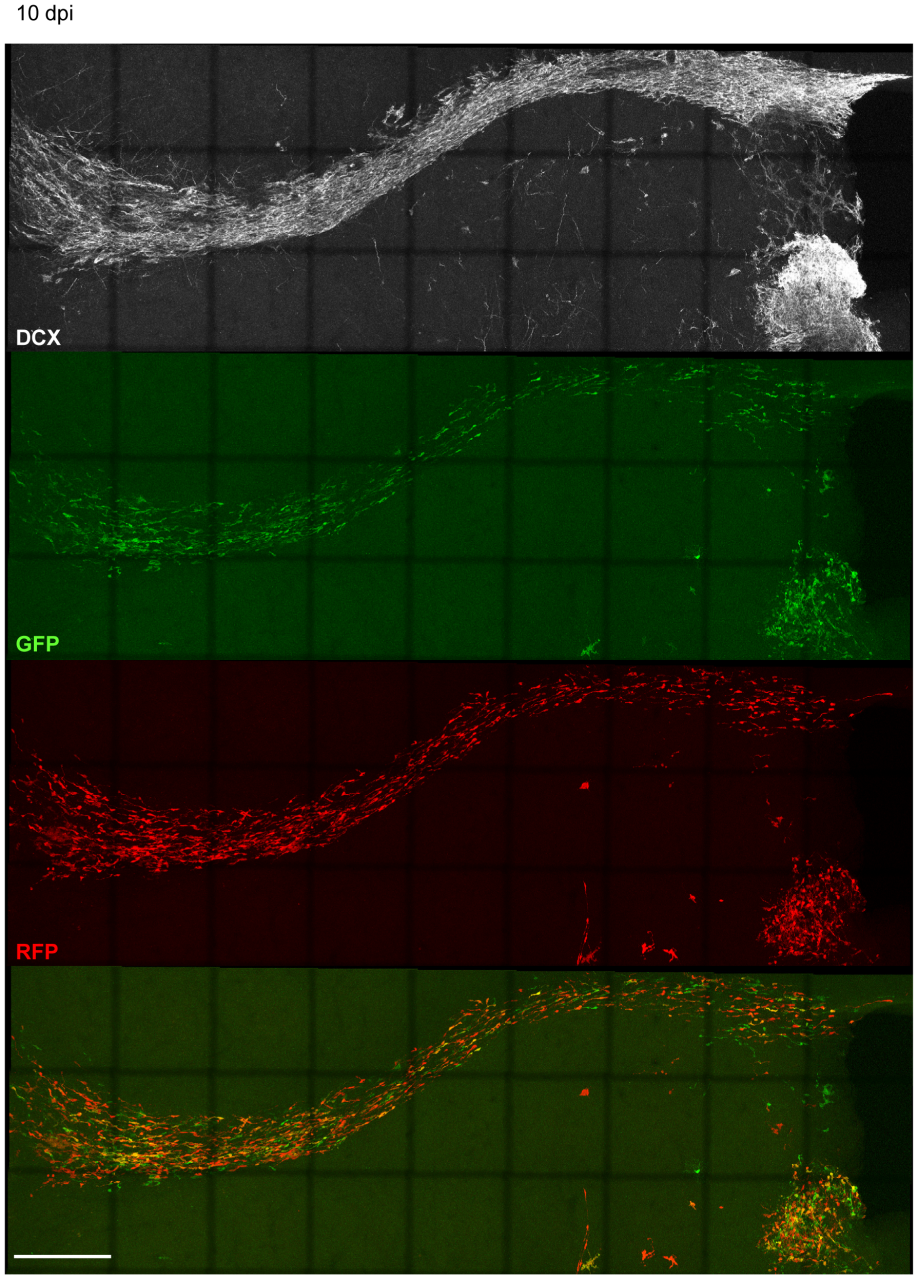
Migration of adult born neurons of the SVZ/OB-system is not altered 10 days after DCX knockdown. DCX ablated neurons (green and yellow) are migrating similar to RFP_only_ control cells at 10 dpi within the RMS.

**Figure 10:**
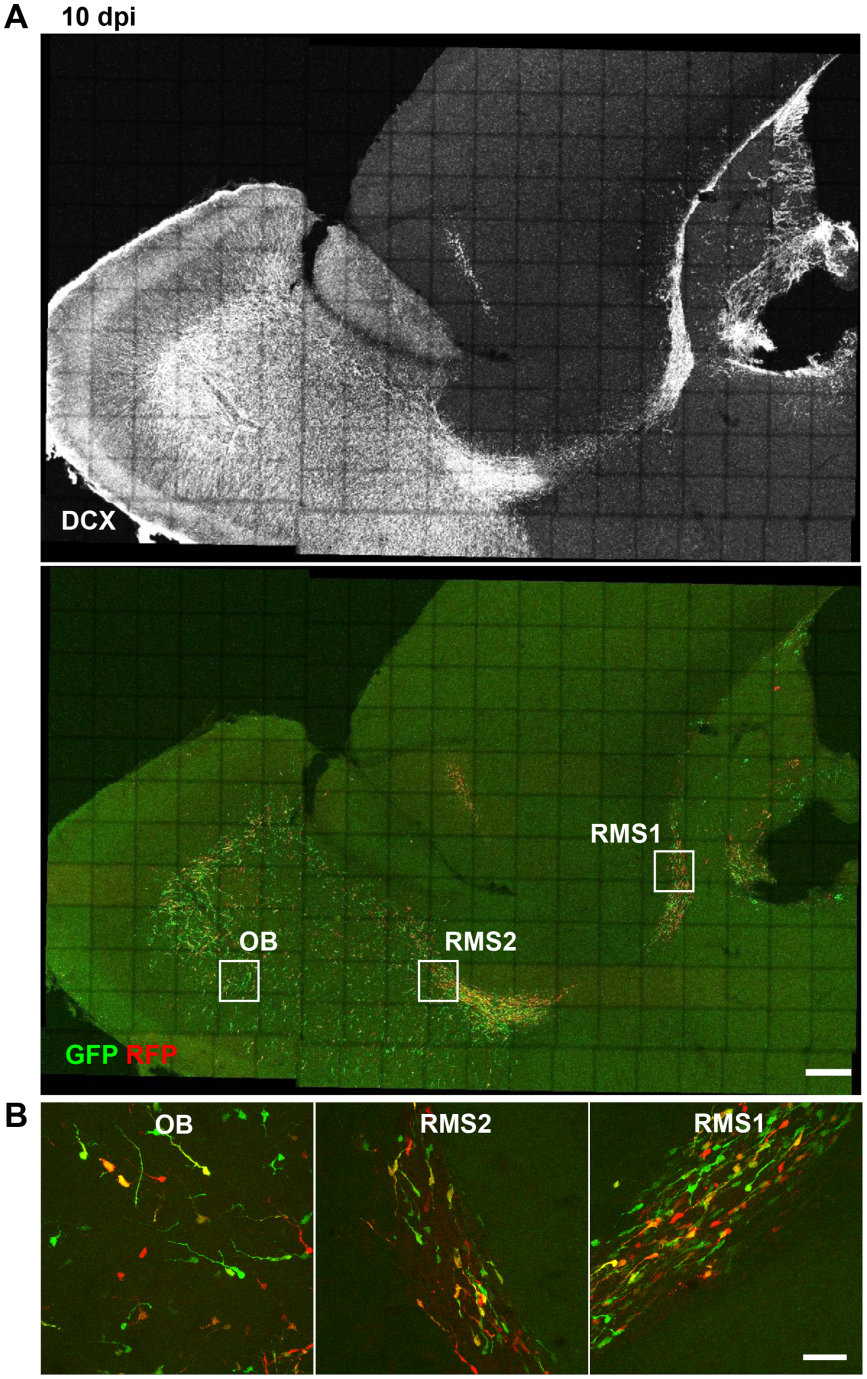
Migration of adult born neurons of the SVZ/OB-system is not altered 10 days after DCX knockdown. Boxes in (A) illustrate the regions (RMS1, RMS2, core OB) that were analysed to determine the relative position of miDCX-transduced cells to control cells. Scale bars 250 µm. (B) The position of miDCX-transduced neurons (green and yellow) to RFP_only_ control neurons (red) is comparable along the RMS/OB at 10 dpi. Scale bar 40 µm.

In the SVZ/OB-system newborn neurons migrate long distances from their place of birth in the SVZ tangentially along the RMS to the OB [32] where they start to migrate radially until they reach their final destination where they differentiate into granule neurons and periglomerular interneurons and integrate into the olfactory network [32], [33]. DCX knockdown in new neurons of the SVZ/OB system causes abnormal neuronal migration and a fate change of developing neurons in the postnatal olfactory bulb 5–7 days post electroporation of the DCX knockdown vector [15]. In DCX null mutants, where DCX is already ablated during development, it has been shown that DCX is needed to maintain the bipolar shape and nuclear translocation during migration [16]. To determine if acute knockdown of DCX in adult born neurons affects their migration, CAG-GFP-miDCX(3) and CAG-RFP-miCtr-IRES-RFP retroviruses were injected into the RMS of 8 week old female wildtype mice (Figure 8A). Like in the DG, DCX is efficiently knocked down in the SVZ (Figure 8B). miDCX(3) and control retrovirus transduced cells did not show apparent differences regarding their position in the RMS/OB system neither at 5 dpi (Figure 8) nor at 10 dpi (Figures 9 and 10). Moreover, RFP_only_ control cells and the green or yellow DCX ablated cells were migrating within the DCX-positive rostral migratory stream (Figures 8, 9, 10). Positioning of DCX-ablated cells relative to control cells was also investigated by determining the ratio of yellow cells to all red cells (yellow+RFP_only_ cells) in three different parts of the RMS/OB system: rostral part of the RMS (RMS1), middle part of the RMS (RMS2), and the core region of the OB where new neurons switch their migratory mode to radial migration (Figure 10). Ratios were comparable between different regions (RMS1∶0.47±0.09; RMS2∶0.55±0.02, OB: 0.48±0.04) confirming that DCX-ablated cells did not assume differential positioning along the dorsoventral axis of the RMS.

**Figure 11:**
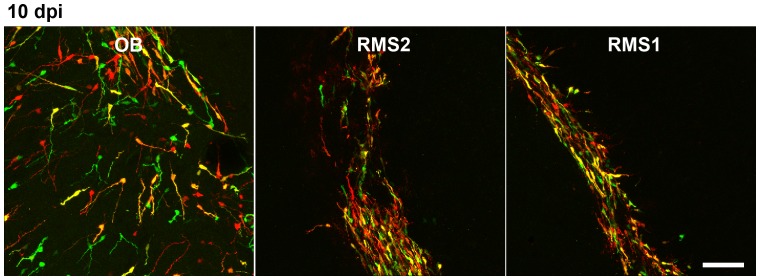
Migration of adult born neurons of the SVZ/OB-system is not altered by DCX overexpression. The position of DCX-overexpressing neurons (green and yellow) to RFP_only_ control neurons (red) is comparable along the RMS/OB at 10 dpi. Scale bar 20 µm.

Finally, the effects of DCX overexpression on migration were investigated during early phases of adult SVZ/OB neurogenesis. Eight weeks old female mice were injected into the RMS with CAG-RFP and CAG-DCX-3xFLAG-IRES-GFP retroviruses and analysed 10 days later. Similar to the knockdown experiments, DCX-overexpressing cells and control transduced cells were found in comparable positions along the RMS/OB system (Ratios RMS1∶0.59±0.12; RMS2∶0.68±0.02, OB: 0.59±0.09) (Figure 11).

Thus, our data indicate that neither knockdown nor overexpression of DCX significantly alter migration in the RMS/OB system at the time points investigated.

## Discussion

The microtubule binding protein DCX is expressed by migrating neuroblasts and immature neurons and is widely used as a stage-specific marker of adult neurogenesis but little is known about its specific function in adult neurogenesis. In this study, we have investigated the consequences of overexpression or knockdown of DCX in adult born neurons of wildtype mice. We did not observe any significant effect on morphological maturation of newly generated DG neurons or on migration of new neurons in either adult neurogenic system at the time points investigated.

For *in vivo* knockdown of DCX we have generated the retroviral CAG-EmGFP-miDCX vector, expressing a miRNA against DCX under the control of an RNA polymerase II promoter. Here we show that our vector has the potential to efficiently knockdown DCX *in vivo* (Figures 5, 6C). By generation of this retroviral miRNA-vector we have created a useful tool for *in vivo* expression of miRNA and knockdown of other proteins of interest.

In DCX knockout mice it was shown that migrating interneurons show branching defects [25]. Here we were investigating if overexpression or knockdown of DCX affected morphological maturation of adult born neurons. Morphological analysis of 10–17 dpi cells was precluded by unequal RFP distribution within the cells. Yet, with an average length of ∼1300 µm and an average number of ∼8 branch points per 10 µm in RFP_only_ control cells and DCX overexpressing or knockdown cells, no morphological defects in the dendritic compartment were apparent at 28 dpi. The relatively low expression levels of GFP precluded reliable axonal tracing of DCX overexpressing or knockdown cells. Thus, it remains to be determined whether DCX gain- and loss-of-function affects development of the axonal compartment.

We found no evidence that DCX gain- and loss-of-function altered migration of new DG neurons from the SGZ towards the granule cell layer. In principle newborn DG cells have the possibility to migrate in three dimensions from their place of birth in the subgranular zone into the granular layer (Figure 2D): medial/lateral (red arrows), septal/temporal (green arrows) and vertically to these two directions through the granular layer (blue arrow). As there is presently no live imaging method established so far to continuously follow the migration of a newborn cell in the dentate gyrus over several weeks, we cannot establish the medial/lateral, septal/temporal position where our retroviral labelled cells are born and cannot determine potential migration deficits along these axes. Hence, it is still formally possible, that DCX – while not being required for migration from the SGZ towards the DG cell layer – may be essential for medial/lateral and septal/temporal migration.

In the SVZ/OB-system newborn neurons migrate long distances from their place of birth in the SVZ tangentially along the RMS to the OB [32] where they start to migrate radially until they reach their final destination and integrate into the olfactory network [32], [33]. DCX is needed for proper migration in the postnatal forebrain, which was shown in juvenile DCX mutant mice (age seven weeks) [16] and by electroporation of shRNAs against DCX into the SVZ of P3/P4 wildtype mice [15], [16]. In contrast to these reports, we did not observe migration defects of newborn neurons of the SVZ/OB-system after retrovirally miRNA-mediated DCX knockdown. In the present study control and manipulating retroviruses were co-injected into the same animal to precisely determine potential migration defects. This strategy allows to compare internal control cells with manipulated cells within one animal, thereby minimizing potential confounding factors, such as variability in the location of injections/electroporations between animals, variability in tissue preparation and generation of equivalent sections, and architectural differences in the SVZ/RMS. Thus, we consider it unlikely, that major migration phenotypes comparable to the phenotypes in the aforementioned studies remained undetected. We, however, cannot exclude that manipulation of DCX will affect later developmental steps of adult SVZ/OB neurogenesis.

The probably most significant difference between our study and the aforementioned studies is the timing of DCX ablation with regard to the age of the mice. In the present study, DCX is knocked down acutely in young adult mice and exclusively in adult born neurons. DCX null mutant mice lack DCX function already during embryonic development and thus do not allow to distinguish whether the observed migratory defects result from a direct function of DCX in adult-born neurons or result from defective CNS development. In the study of Belvindrah and colleagues [15], DCX is knocked down at P4. Early postnatal neurogenesis and adult neurogenesis have been found to generate morphologically and functionally distinct neuronal population for the OB neural circuit [34]. It is therefore possible that the obvious difference between our findings and the findings of Belvindrah and colleagues reflect an age-dependent switch in the function of DCX in neuronal migration in the SVZ/OB system.

A second significant difference from the study of Belvindrah and colleagues is, that in our study, miDCX-retroviruses were injected into the RMS and not the SVZ. These targeting strategies may hit different population of newborn neurons, as dopaminergic periglomerular neurons are predominantly generated in the RMS [35] while granule neurons are generated in the SVZ. Moreover, electroporation into the SVZ may have predominantly targeted early precursors of the SVZ prior to initiation of DCX expression, whereas our strategy may have targeted dividing neuroblasts in the RMS, which already initiated DCX expression and migration. Thus, it is possible that in the adult mouse brain, DCX may be required solely for initiation rather than sustenance of migration.

At present, it remains unclear, how adult-born neurons compensated for the extensive loss of DCX. We cannot exclude that ad libitum access to running wheels, which in the context of the low titer miRNA vector was necessary to increase the number of transduced cells, may enhance the expression of factors that render newly generated born neurons resistant to DCX loss-of-function. A second possibility would be that smallest amounts of remaining DCX expression following knockdown, which escape detection by immunstainings, may be sufficient for proper maturation of the cells. In our view, the most likely explanation would be that the doublecortin like kinase (DCLK), which is closely related to DCX, has redundant function to DCX during murine cortical [36], [37], [38] and hippocampal development [39] and is co-expressed with DCX in murine adult neurogenic niches [40], might be sufficient to regulate proper development of newborn neurons following efficient DCX knockdown.

Generation of DCX conditional knockout mice will help to resolve these open questions and will greatly facilitate the future investigation of potentially hidden functions of DCX in adult neurogenesis.
